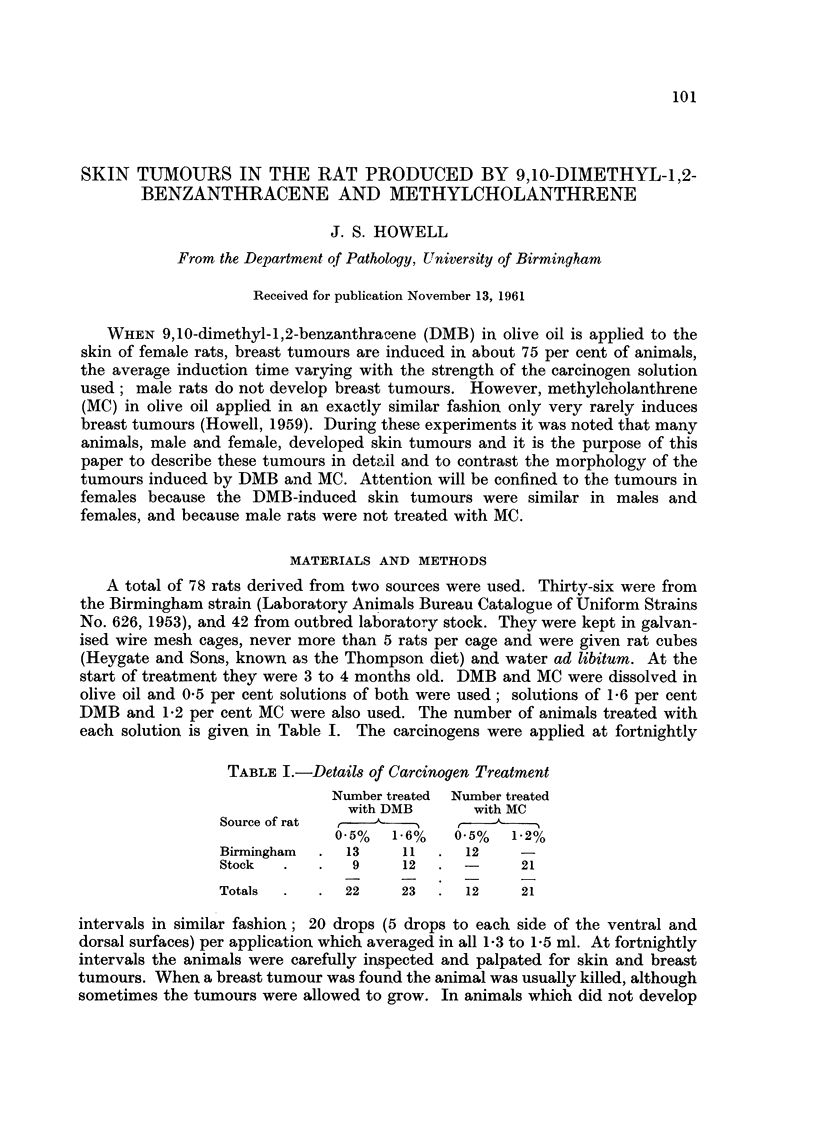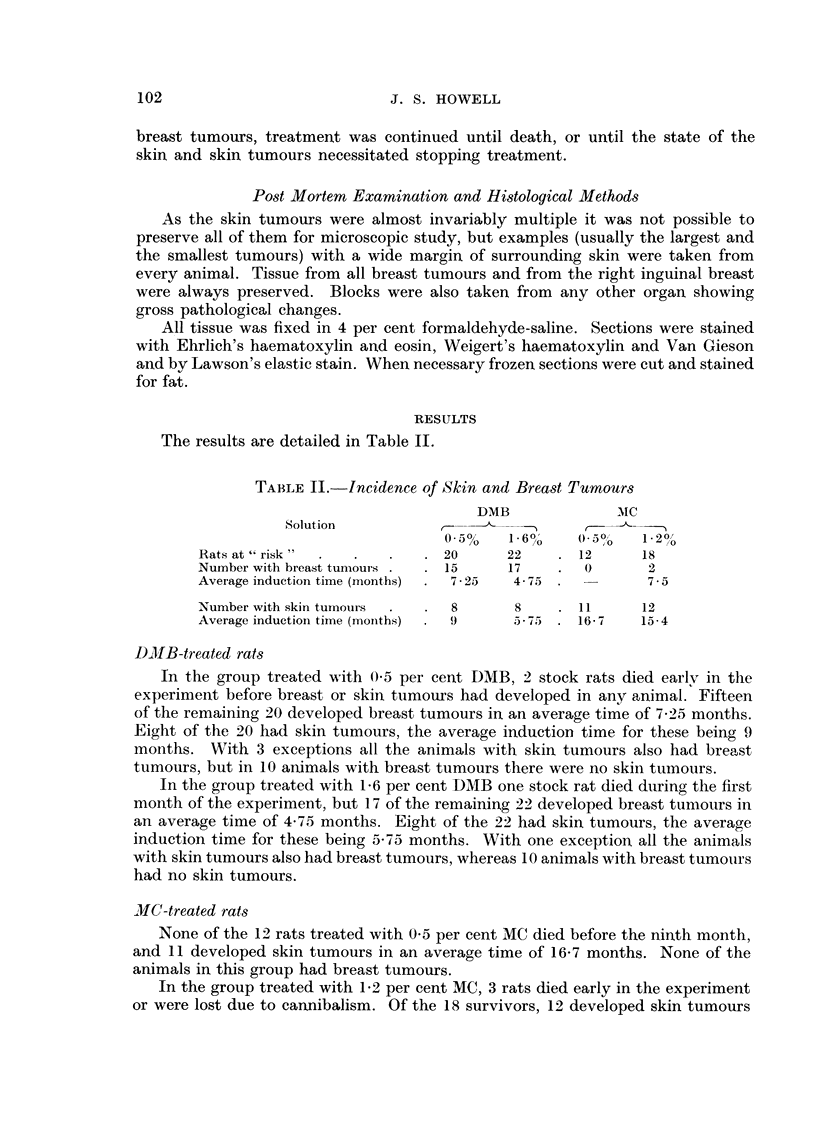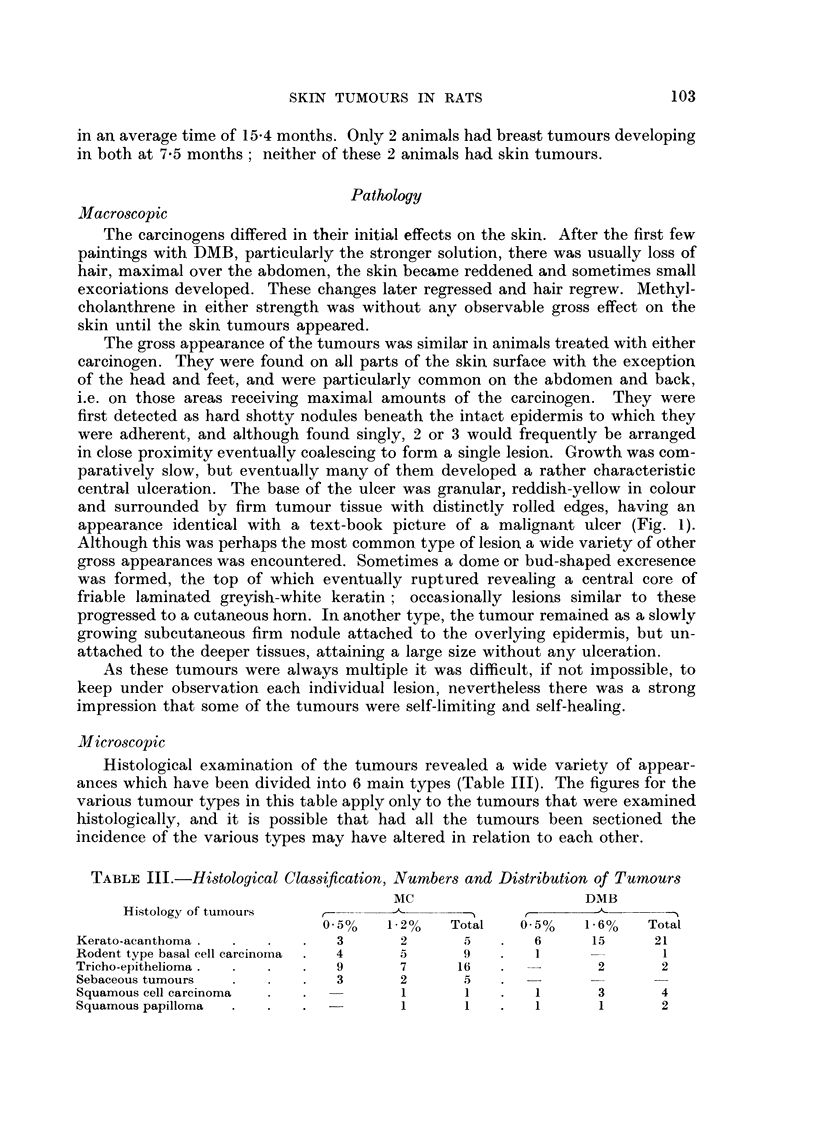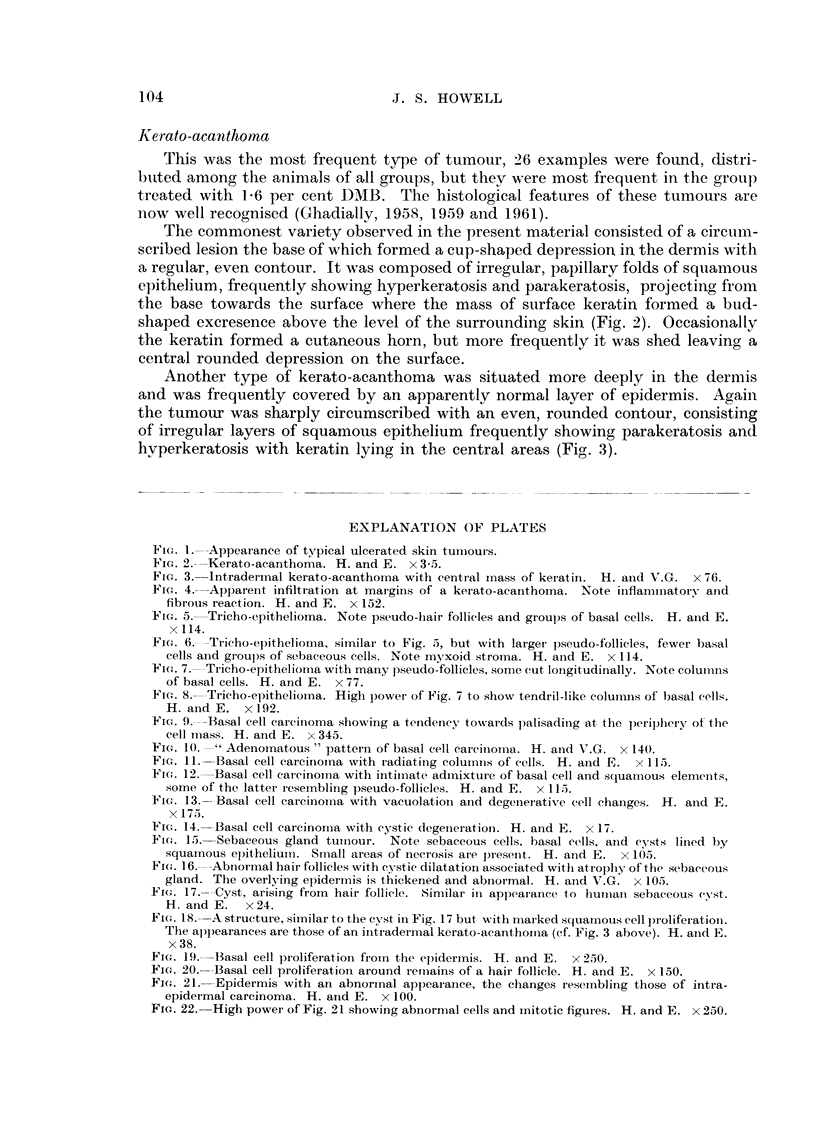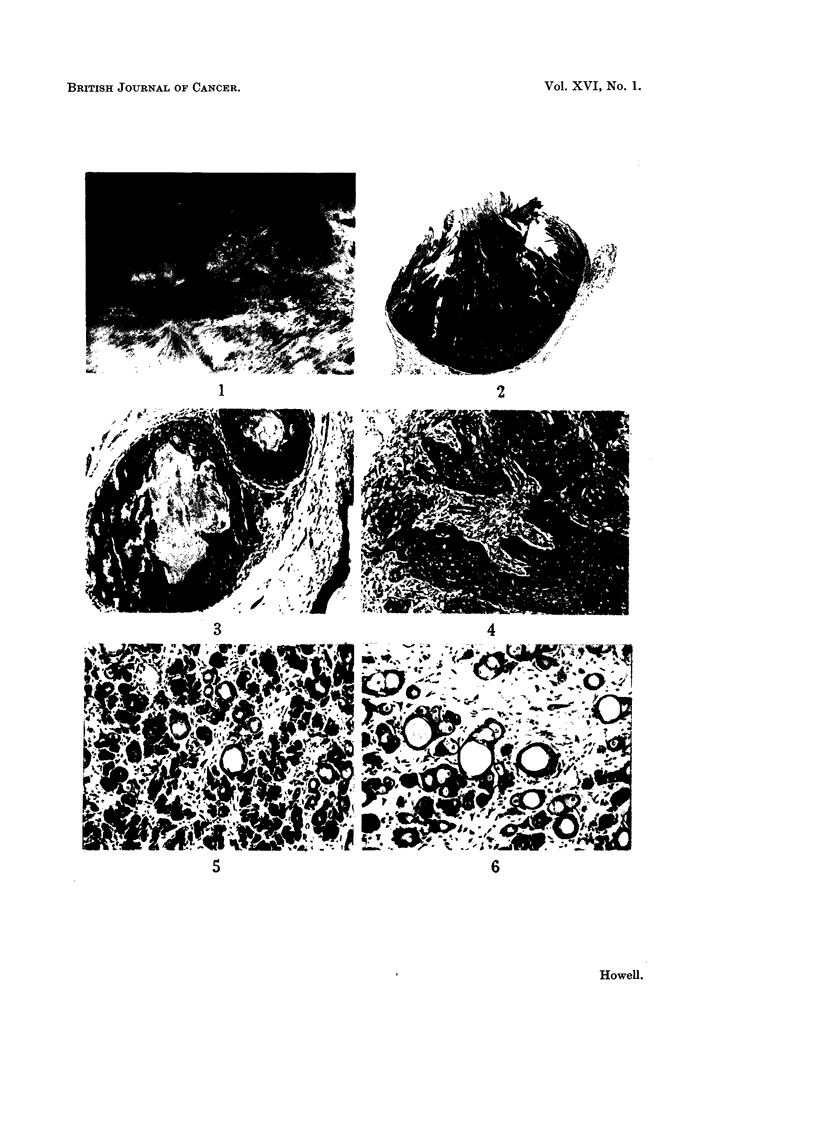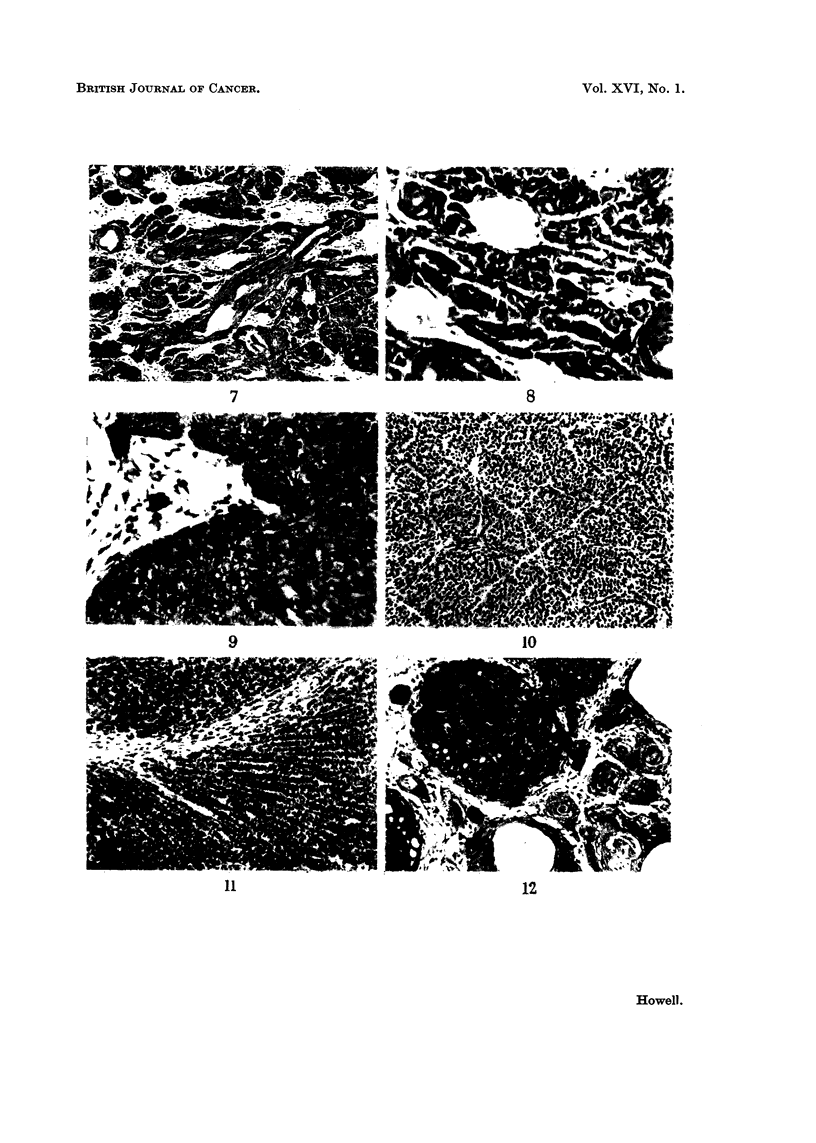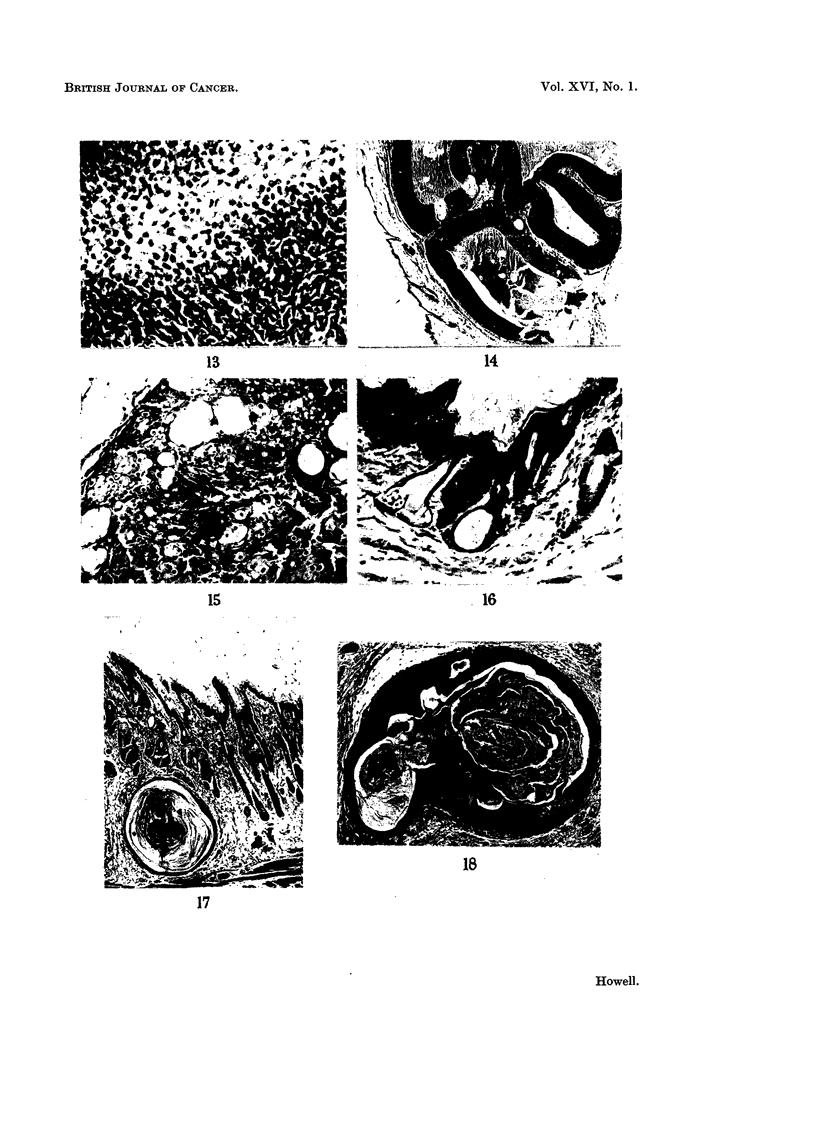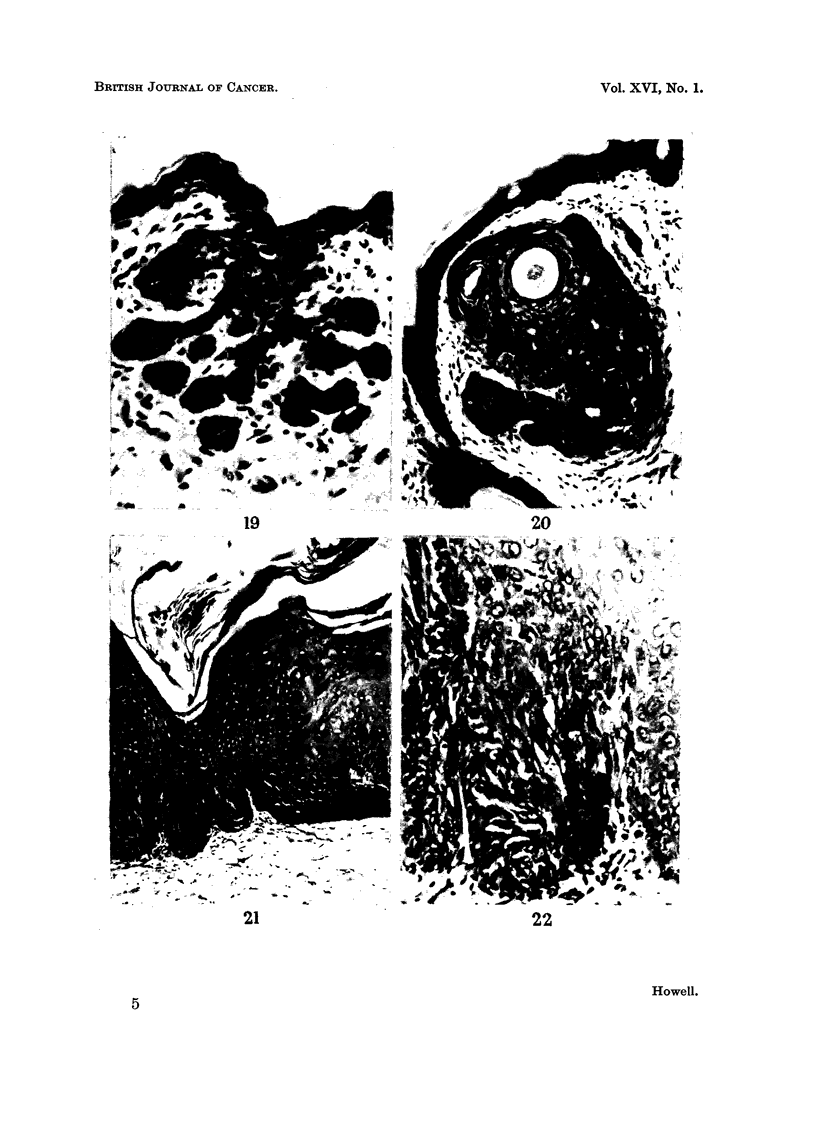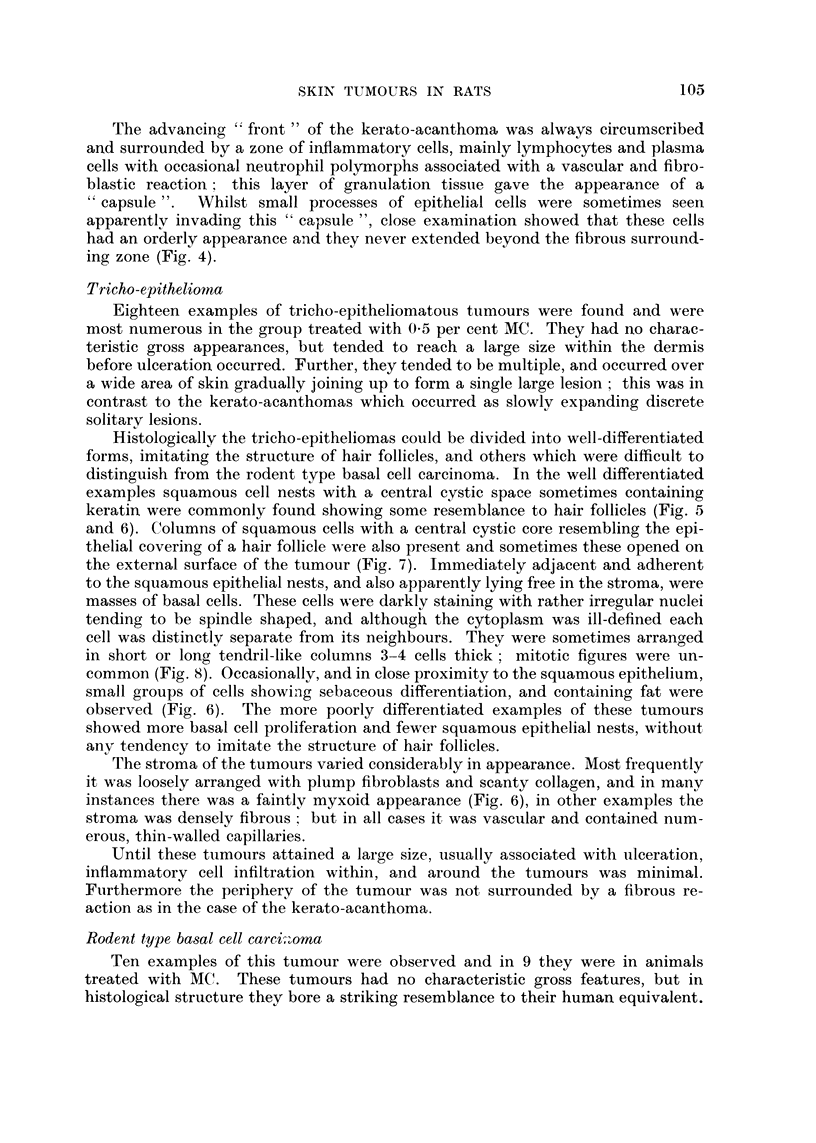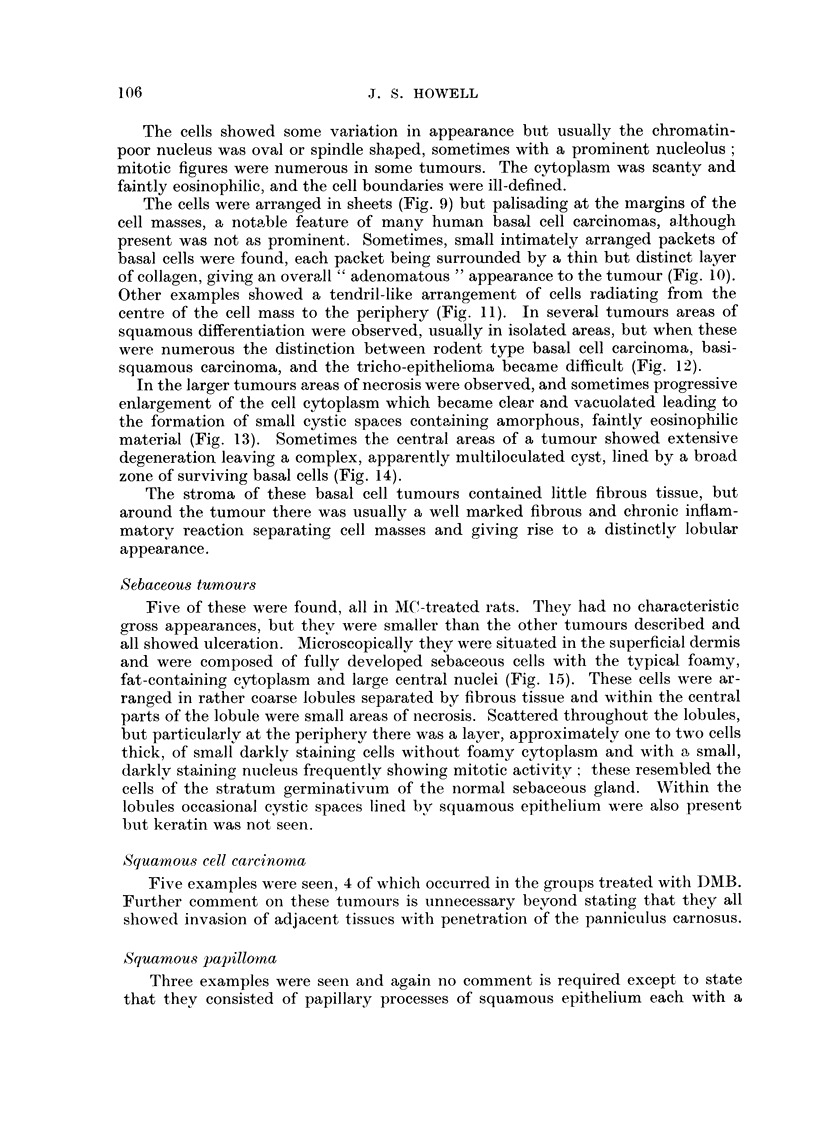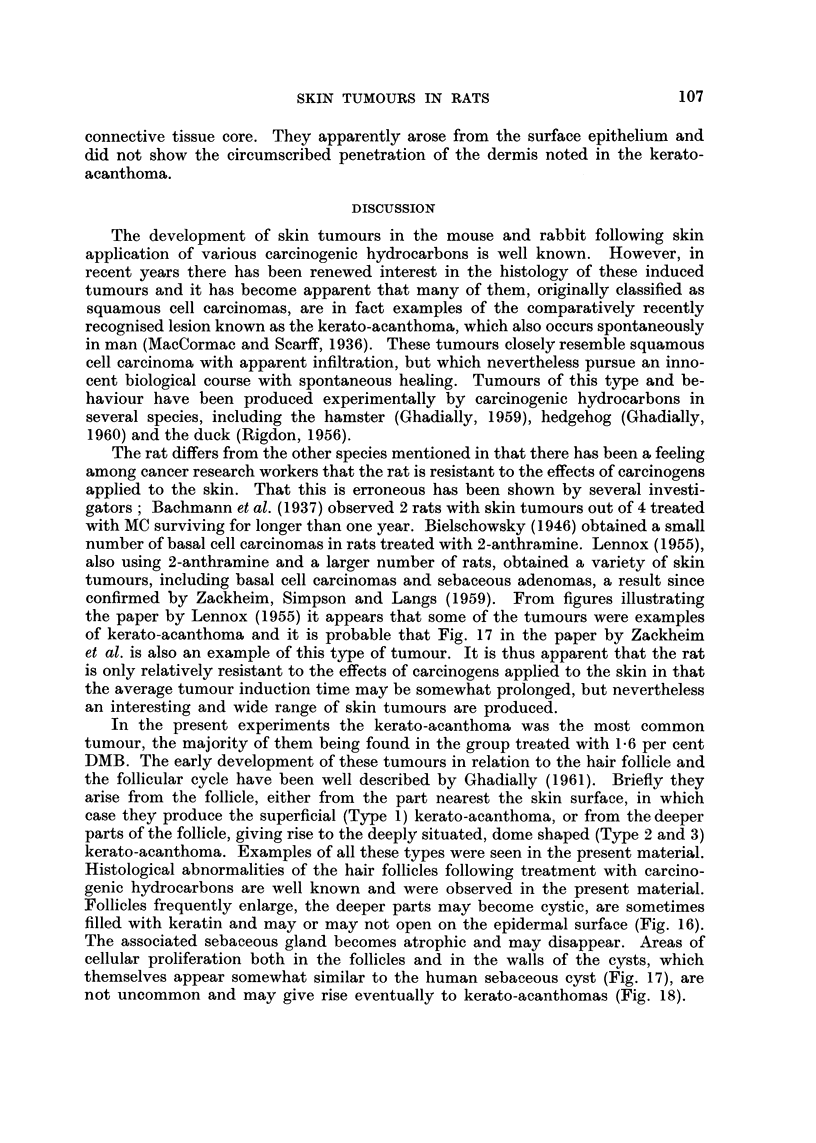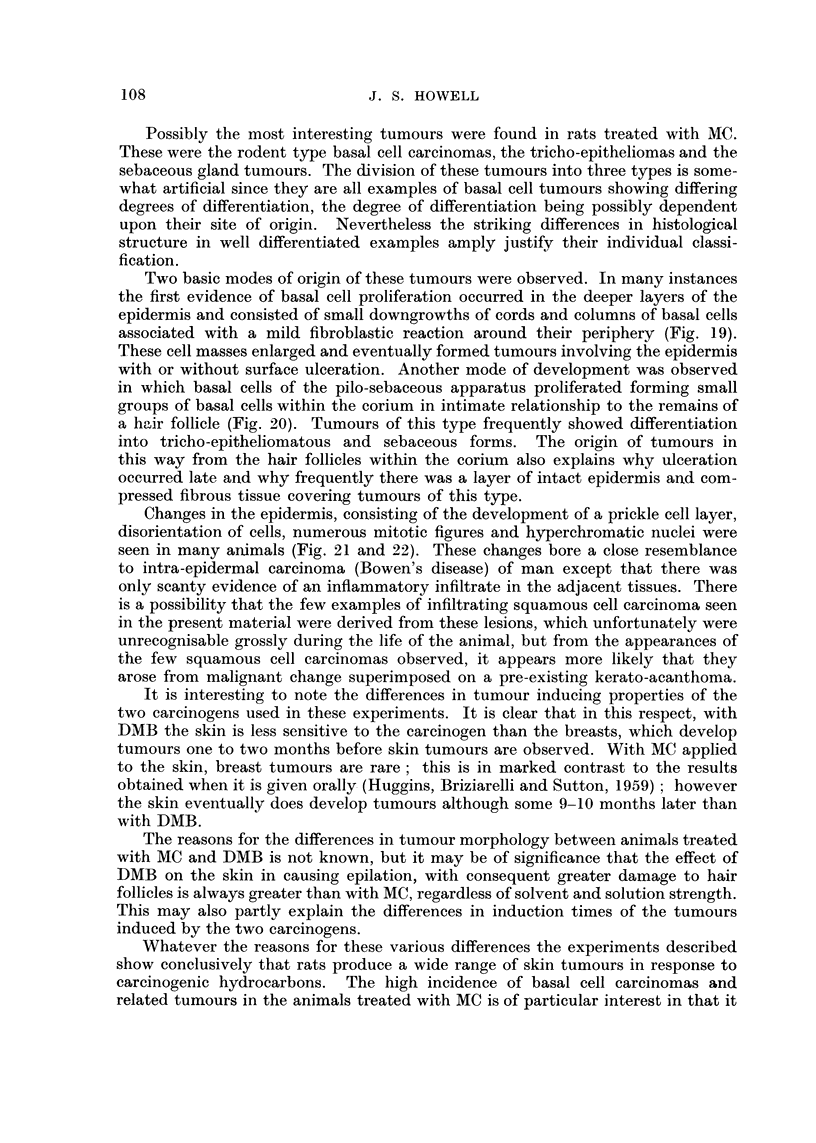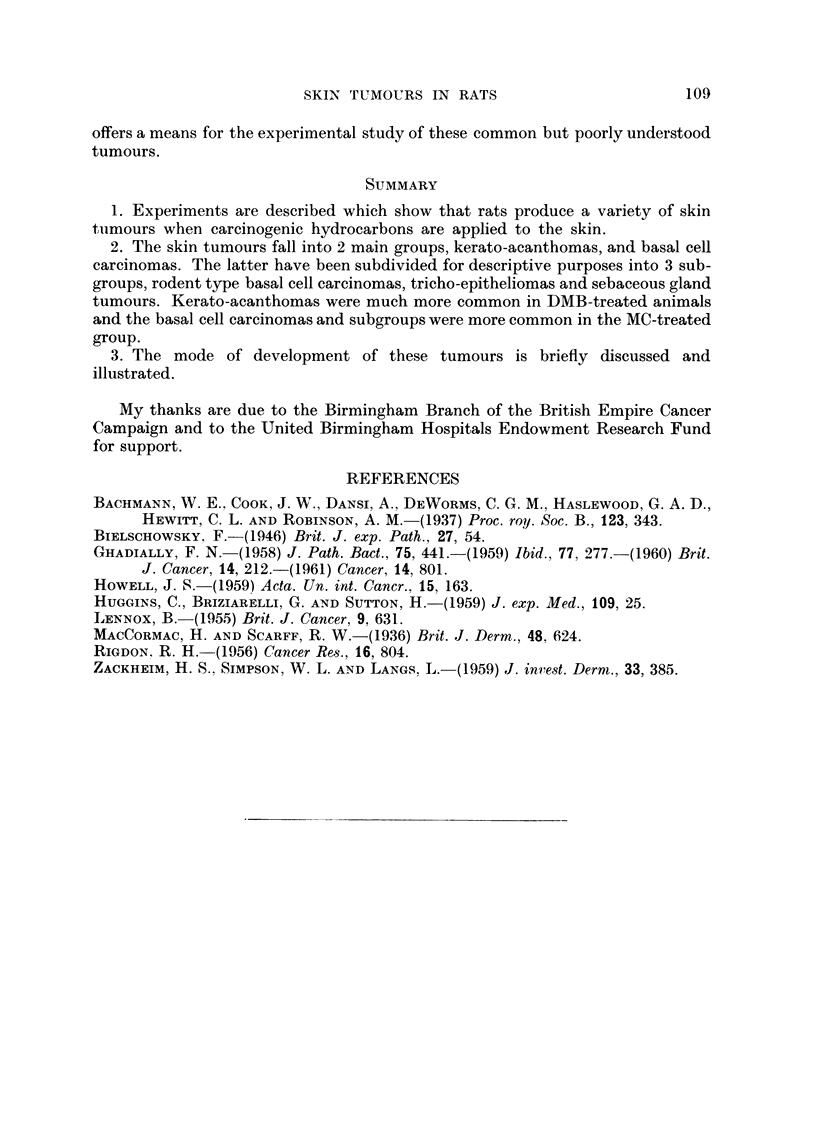# Skin Tumours in the Rat Produced by 9,10-Dimethyl-1,2-Benzanthracene and Methylcholanthrene

**DOI:** 10.1038/bjc.1962.10

**Published:** 1962-03

**Authors:** J. S. Howell

## Abstract

**Images:**


### SKIN TUMOURS IN THE RAT PRODUCED BY 9,10-DIMETHYL-1,2-BENZANTHRACENE AND METHYLCHOLANTHRENE

J. S. HOWELL

*From the Department of Pathology, University of Birmingham*

Received for publication November 13, 1961

WHEN 9,10-dimethyl-1,2-benzanthracene (DMB) in olive oil is applied to the skin of female rats, breast tumours are induced in about 75 per cent of animals, the average induction time varying with the strength of the carcinogen solution used ; male rats do not develop breast tumours. However, methylcholanthrene (MC) in olive oil applied in an exactly similar fashion only very rarely induces breast tumours (Howell, 1959). During these experiments it was noted that many animals, male and female, developed skin tumours and it is the purpose of this paper to describe these tumours in detail and to contrast the morphology of the tumours induced by DMB and MC. Attention will be confined to the tumours in females because the DMB-induced skin tumours were similar in males and females, and because male rats were not treated with MC.

### MATERIALS AND METHODS

A total of 78 rats derived from two sources were used. Thirty-six were from the Birmingham strain (Laboratory Animals Bureau Catalogue of Uniform Strains No. 626, 1953), and 42 from outbred laboratory stock. They were kept in galvanised wire mesh cages, never more than 5 rats per cage and were given rat cubes (Heygate and Sons, known as the Thompson diet) and water *ad libitum*. At the start of treatment they were 3 to 4 months old. DMB and MC were dissolved in olive oil and 0·5 per cent solutions of both were used ; solutions of 1·6 per cent DMB and 1·2 per cent MC were also used. The number of animals treated with each solution is given in Table I. The carcinogens were applied at fortnightly intervals in similar fashion ; 20 drops (5 drops to each side of the ventral and dorsal surfaces) per application which averaged in all 1·3 to 1·5 ml. At fortnightly intervals the animals were carefully inspected and palpated for skin and breast tumours. When a breast tumour was found the animal was usually killed, although sometimes the tumours were allowed to grow. In animals which did not develop

TABLE I.—*Details of Carcinogen Treatment*

| Source of rat | Number treated with DMB | | Number treated with MC | |
|---|---|---|---|---|
| | 0·5% | 1·6% | 0·5% | 1·2% |
| Birmingham | 13 | 11 | 12 | — |
| Stock | 9 | 12 | — | 21 |
| Totals | 22 | 23 | 12 | 21 |

breast tumours, treatment was continued until death, or until the state of the skin and skin tumours necessitated stopping treatment.

### *Post Mortem Examination and Histological Methods*

As the skin tumours were almost invariably multiple it was not possible to preserve all of them for microscopic study, but examples (usually the largest and the smallest tumours) with a wide margin of surrounding skin were taken from every animal. Tissue from all breast tumours and from the right inguinal breast were always preserved. Blocks were also taken from any other organ showing gross pathological changes.

All tissue was fixed in 4 per cent formaldehyde-saline. Sections were stained with Ehrlich's haematoxylin and eosin, Weigert's haematoxylin and Van Gieson and by Lawson's elastic stain. When necessary frozen sections were cut and stained for fat.

### RESULTS

The results are detailed in Table II.

TABLE II.—*Incidence of Skin and Breast Tumours*

| Solution | DMB | | MC | |
|---|---|---|---|---|
| | 0·5% | 1·6% | 0·5% | 1·2% |
| Rats at " risk " | 20 | 22 | 12 | 18 |
| Number with breast tumours | 15 | 17 | 0 | 2 |
| Average induction time (months) | 7·25 | 4·75 | — | 7·5 |
| Number with skin tumours | 8 | 8 | 11 | 12 |
| Average induction time (months) | 9 | 5·75 | 16·7 | 15·4 |

### *DMB-treated rats*

In the group treated with 0·5 per cent DMB, 2 stock rats died early in the experiment before breast or skin tumours had developed in any animal. Fifteen of the remaining 20 developed breast tumours in an average time of 7·25 months. Eight of the 20 had skin tumours, the average induction time for these being 9 months. With 3 exceptions all the animals with skin tumours also had breast tumours, but in 10 animals with breast tumours there were no skin tumours.

In the group treated with 1·6 per cent DMB one stock rat died during the first month of the experiment, but 17 of the remaining 22 developed breast tumours in an average time of 4·75 months. Eight of the 22 had skin tumours, the average induction time for these being 5·75 months. With one exception all the animals with skin tumours also had breast tumours, whereas 10 animals with breast tumours had no skin tumours.

### *MC-treated rats*

None of the 12 rats treated with 0·5 per cent MC died before the ninth month, and 11 developed skin tumours in an average time of 16·7 months. None of the animals in this group had breast tumours.

In the group treated with 1·2 per cent MC, 3 rats died early in the experiment or were lost due to cannibalism. Of the 18 survivors, 12 developed skin tumours in an average time of 15·4 months. Only 2 animals had breast tumours developing in both at 7·5 months ; neither of these 2 animals had skin tumours.

### *Pathology*

### *Macroscopic*

The carcinogens differed in their initial effects on the skin. After the first few paintings with DMB, particularly the stronger solution, there was usually loss of hair, maximal over the abdomen, the skin became reddened and sometimes small excoriations developed. These changes later regressed and hair regrew. Methylcholanthrene in either strength was without any observable gross effect on the skin until the skin tumours appeared.

The gross appearance of the tumours was similar in animals treated with either carcinogen. They were found on all parts of the skin surface with the exception of the head and feet, and were particularly common on the abdomen and back, i.e. on those areas receiving maximal amounts of the carcinogen. They were first detected as hard shotty nodules beneath the intact epidermis to which they were adherent, and although found singly, 2 or 3 would frequently be arranged in close proximity eventually coalescing to form a single lesion. Growth was comparatively slow, but eventually many of them developed a rather characteristic central ulceration. The base of the ulcer was granular, reddish-yellow in colour and surrounded by firm tumour tissue with distinctly rolled edges, having an appearance identical with a text-book picture of a malignant ulcer (Fig. 1). Although this was perhaps the most common type of lesion a wide variety of other gross appearances was encountered. Sometimes a dome or bud-shaped excresence was formed, the top of which eventually ruptured revealing a central core of friable laminated greyish-white keratin ; occasionally lesions similar to these progressed to a cutaneous horn. In another type, the tumour remained as a slowly growing subcutaneous firm nodule attached to the overlying epidermis, but unattached to the deeper tissues, attaining a large size without any ulceration.

As these tumours were always multiple it was difficult, if not impossible, to keep under observation each individual lesion, nevertheless there was a strong impression that some of the tumours were self-limiting and self-healing.

### *Microscopic*

Histological examination of the tumours revealed a wide variety of appearances which have been divided into 6 main types (Table III). The figures for the various tumour types in this table apply only to the tumours that were examined histologically, and it is possible that had all the tumours been sectioned the incidence of the various types may have altered in relation to each other.

TABLE III.—*Histological Classification, Numbers and Distribution of Tumours*

| Histology of tumours | MC | | | DMB | | |
|---|---|---|---|---|---|---|
| | 0·5% | 1·2% | Total | 0·5% | 1·6% | Total |
| Kerato-acanthoma | 3 | 2 | 5 | 6 | 15 | 21 |
| Rodent type basal cell carcinoma | 4 | 5 | 9 | 1 | — | 1 |
| Tricho-epithelioma | 9 | 7 | 16 | — | 2 | 2 |
| Sebaceous tumours | 3 | 2 | 5 | — | — | — |
| Squamous cell carcinoma | — | 1 | 1 | 1 | 3 | 4 |
| Squamous papilloma | — | 1 | 1 | 1 | 1 | 2 |

*Kerato-acanthoma*

This was the most frequent type of tumour, 26 examples were found, distributed among the animals of all groups, but they were most frequent in the group treated with 1·6 per cent DMB. The histological features of these tumours are now well recognised (Ghadially, 1958, 1959 and 1961).

The commonest variety observed in the present material consisted of a circumscribed lesion the base of which formed a cup-shaped depression in the dermis with a regular, even contour. It was composed of irregular, papillary folds of squamous epithelium, frequently showing hyperkeratosis and parakeratosis, projecting from the base towards the surface where the mass of surface keratin formed a bud-shaped excresence above the level of the surrounding skin (Fig. 2). Occasionally the keratin formed a cutaneous horn, but more frequently it was shed leaving a central rounded depression on the surface.

Another type of kerato-acanthoma was situated more deeply in the dermis and was frequently covered by an apparently normal layer of epidermis. Again the tumour was sharply circumscribed with an even, rounded contour, consisting of irregular layers of squamous epithelium frequently showing parakeratosis and hyperkeratosis with keratin lying in the central areas (Fig. 3).

---

EXPLANATION OF PLATES

FIG. 1.—Appearance of typical ulcerated skin tumours.

FIG. 2.—Kerato-acanthoma. H. and E. ×3·5.

FIG. 3.—Intradermal kerato-acanthoma with central mass of keratin. H. and V.G. ×76.

FIG. 4.—Apparent infiltration at margins of a kerato-acanthoma. Note inflammatory and fibrous reaction. H. and E. ×152.

FIG. 5.—Tricho-epithelioma. Note pseudo-hair follicles and groups of basal cells. H. and E. ×114.

FIG. 6.—Tricho-epithelioma, similar to Fig. 5, but with larger pseudo-follicles, fewer basal cells and groups of sebaceous cells. Note myxoid stroma. H. and E. ×114.

FIG. 7.—Tricho-epithelioma with many pseudo-follicles, some cut longitudinally. Note columns of basal cells. H. and E. ×77.

FIG. 8.—Tricho-epithelioma. High power of Fig. 7 to show tendril-like columns of basal cells. H. and E. ×192.

FIG. 9.—Basal cell carcinoma showing a tendency towards palisading at the periphery of the cell mass. H. and E. ×345.

FIG. 10.—" Adenomatous " pattern of basal cell carcinoma. H. and V.G. ×140.

FIG. 11.—Basal cell carcinoma with radiating columns of cells. H. and E. ×115.

FIG. 12.—Basal cell carcinoma with intimate admixture of basal cell and squamous elements, some of the latter resembling pseudo-follicles. H. and E. ×115.

FIG. 13.—Basal cell carcinoma with vacuolation and degenerative cell changes. H. and E. ×175.

FIG. 14.—Basal cell carcinoma with cystic degeneration. H. and E. ×17.

FIG. 15.—Sebaceous gland tumour. Note sebaceous cells, basal cells, and cysts lined by squamous epithelium. Small areas of necrosis are present. H. and E. ×105.

FIG. 16.—Abnormal hair follicles with cystic dilatation associated with atrophy of the sebaceous gland. The overlying epidermis is thickened and abnormal. H. and V.G. ×105.

FIG. 17.—Cyst, arising from hair follicle. Similar in appearance to human sebaceous cyst. H. and E. ×24.

FIG. 18.—A structure, similar to the cyst in Fig. 17 but with marked squamous cell proliferation. The appearances are those of an intradermal kerato-acanthoma (cf. Fig. 3 above). H. and E. ×38.

FIG. 19.—Basal cell proliferation from the epidermis. H. and E. ×250.

FIG. 20.—Basal cell proliferation around remains of a hair follicle. H. and E. ×150.

FIG. 21.—Epidermis with an abnormal appearance, the changes resembling those of intra-epidermal carcinoma. H. and E. ×100.

FIG. 22.—High power of Fig. 21 showing abnormal cells and mitotic figures. H. and E. ×250.

1

2

3

4

5

6

7

8

9

10

11

12

Howell.

13

14

15

16

17

18

19

20

21

22

The advancing " front " of the kerato-acanthoma was always circumscribed and surrounded by a zone of inflammatory cells, mainly lymphocytes and plasma cells with occasional neutrophil polymorphs associated with a vascular and fibroblastic reaction ; this layer of granulation tissue gave the appearance of a " capsule ". Whilst small processes of epithelial cells were sometimes seen apparently invading this " capsule ", close examination showed that these cells had an orderly appearance and they never extended beyond the fibrous surrounding zone (Fig. 4).

*Tricho-epithelioma*

Eighteen examples of tricho-epitheliomatous tumours were found and were most numerous in the group treated with 0·5 per cent MC. They had no characteristic gross appearances, but tended to reach a large size within the dermis before ulceration occurred. Further, they tended to be multiple, and occurred over a wide area of skin gradually joining up to form a single large lesion ; this was in contrast to the kerato-acanthomas which occurred as slowly expanding discrete solitary lesions.

Histologically the tricho-epitheliomas could be divided into well-differentiated forms, imitating the structure of hair follicles, and others which were difficult to distinguish from the rodent type basal cell carcinoma. In the well differentiated examples squamous cell nests with a central cystic space sometimes containing keratin were commonly found showing some resemblance to hair follicles (Fig. 5 and 6). Columns of squamous cells with a central cystic core resembling the epithelial covering of a hair follicle were also present and sometimes these opened on the external surface of the tumour (Fig. 7). Immediately adjacent and adherent to the squamous epithelial nests, and also apparently lying free in the stroma, were masses of basal cells. These cells were darkly staining with rather irregular nuclei tending to be spindle shaped, and although the cytoplasm was ill-defined each cell was distinctly separate from its neighbours. They were sometimes arranged in short or long tendril-like columns 3–4 cells thick ; mitotic figures were uncommon (Fig. 8). Occasionally, and in close proximity to the squamous epithelium, small groups of cells showing sebaceous differentiation, and containing fat were observed (Fig. 6). The more poorly differentiated examples of these tumours showed more basal cell proliferation and fewer squamous epithelial nests, without any tendency to imitate the structure of hair follicles.

The stroma of the tumours varied considerably in appearance. Most frequently it was loosely arranged with plump fibroblasts and scanty collagen, and in many instances there was a faintly myxoid appearance (Fig. 6), in other examples the stroma was densely fibrous ; but in all cases it was vascular and contained numerous, thin-walled capillaries.

Until these tumours attained a large size, usually associated with ulceration, inflammatory cell infiltration within, and around the tumours was minimal. Furthermore the periphery of the tumour was not surrounded by a fibrous reaction as in the case of the kerato-acanthoma.

*Rodent type basal cell carcinoma*

Ten examples of this tumour were observed and in 9 they were in animals treated with MC. These tumours had no characteristic gross features, but in histological structure they bore a striking resemblance to their human equivalent.

The cells showed some variation in appearance but usually the chromatin-poor nucleus was oval or spindle shaped, sometimes with a prominent nucleolus; mitotic figures were numerous in some tumours. The cytoplasm was scanty and faintly eosinophilic, and the cell boundaries were ill-defined.

The cells were arranged in sheets (Fig. 9) but palisading at the margins of the cell masses, a notable feature of many human basal cell carcinomas, although present was not as prominent. Sometimes, small intimately arranged packets of basal cells were found, each packet being surrounded by a thin but distinct layer of collagen, giving an overall " adenomatous " appearance to the tumour (Fig. 10). Other examples showed a tendril-like arrangement of cells radiating from the centre of the cell mass to the periphery (Fig. 11). In several tumours areas of squamous differentiation were observed, usually in isolated areas, but when these were numerous the distinction between rodent type basal cell carcinoma, basi-squamous carcinoma, and the tricho-epithelioma became difficult (Fig. 12).

In the larger tumours areas of necrosis were observed, and sometimes progressive enlargement of the cell cytoplasm which became clear and vacuolated leading to the formation of small cystic spaces containing amorphous, faintly eosinophilic material (Fig. 13). Sometimes the central areas of a tumour showed extensive degeneration leaving a complex, apparently multiloculated cyst, lined by a broad zone of surviving basal cells (Fig. 14).

The stroma of these basal cell tumours contained little fibrous tissue, but around the tumour there was usually a well marked fibrous and chronic inflammatory reaction separating cell masses and giving rise to a distinctly lobular appearance.

*Sebaceous tumours*

Five of these were found, all in MC-treated rats. They had no characteristic gross appearances, but they were smaller than the other tumours described and all showed ulceration. Microscopically they were situated in the superficial dermis and were composed of fully developed sebaceous cells with the typical foamy, fat-containing cytoplasm and large central nuclei (Fig. 15). These cells were arranged in rather coarse lobules separated by fibrous tissue and within the central parts of the lobule were small areas of necrosis. Scattered throughout the lobules, but particularly at the periphery there was a layer, approximately one to two cells thick, of small darkly staining cells without foamy cytoplasm and with a small, darkly staining nucleus frequently showing mitotic activity; these resembled the cells of the stratum germinativum of the normal sebaceous gland. Within the lobules occasional cystic spaces lined by squamous epithelium were also present but keratin was not seen.

*Squamous cell carcinoma*

Five examples were seen, 4 of which occurred in the groups treated with DMB. Further comment on these tumours is unnecessary beyond stating that they all showed invasion of adjacent tissues with penetration of the panniculus carnosus.

*Squamous papilloma*

Three examples were seen and again no comment is required except to state that they consisted of papillary processes of squamous epithelium each with a connective tissue core. They apparently arose from the surface epithelium and did not show the circumscribed penetration of the dermis noted in the kerato-acanthoma.

### DISCUSSION

The development of skin tumours in the mouse and rabbit following skin application of various carcinogenic hydrocarbons is well known. However, in recent years there has been renewed interest in the histology of these induced tumours and it has become apparent that many of them, originally classified as squamous cell carcinomas, are in fact examples of the comparatively recently recognised lesion known as the kerato-acanthoma, which also occurs spontaneously in man (MacCormac and Scarff, 1936). These tumours closely resemble squamous cell carcinoma with apparent infiltration, but which nevertheless pursue an innocent biological course with spontaneous healing. Tumours of this type and behaviour have been produced experimentally by carcinogenic hydrocarbons in several species, including the hamster (Ghadially, 1959), hedgehog (Ghadially, 1960) and the duck (Rigdon, 1956).

The rat differs from the other species mentioned in that there has been a feeling among cancer research workers that the rat is resistant to the effects of carcinogens applied to the skin. That this is erroneous has been shown by several investigators ; Bachmann *et al.* (1937) observed 2 rats with skin tumours out of 4 treated with MC surviving for longer than one year. Bielschowsky (1946) obtained a small number of basal cell carcinomas in rats treated with 2-anthramine. Lennox (1955), also using 2-anthramine and a larger number of rats, obtained a variety of skin tumours, including basal cell carcinomas and sebaceous adenomas, a result since confirmed by Zackheim, Simpson and Langs (1959). From figures illustrating the paper by Lennox (1955) it appears that some of the tumours were examples of kerato-acanthoma and it is probable that Fig. 17 in the paper by Zackheim *et al.* is also an example of this type of tumour. It is thus apparent that the rat is only relatively resistant to the effects of carcinogens applied to the skin in that the average tumour induction time may be somewhat prolonged, but nevertheless an interesting and wide range of skin tumours are produced.

In the present experiments the kerato-acanthoma was the most common tumour, the majority of them being found in the group treated with 1·6 per cent DMB. The early development of these tumours in relation to the hair follicle and the follicular cycle have been well described by Ghadially (1961). Briefly they arise from the follicle, either from the part nearest the skin surface, in which case they produce the superficial (Type 1) kerato-acanthoma, or from the deeper parts of the follicle, giving rise to the deeply situated, dome shaped (Type 2 and 3) kerato-acanthoma. Examples of all these types were seen in the present material. Histological abnormalities of the hair follicles following treatment with carcinogenic hydrocarbons are well known and were observed in the present material. Follicles frequently enlarge, the deeper parts may become cystic, are sometimes filled with keratin and may or may not open on the epidermal surface (Fig. 16). The associated sebaceous gland becomes atrophic and may disappear. Areas of cellular proliferation both in the follicles and in the walls of the cysts, which themselves appear somewhat similar to the human sebaceous cyst (Fig. 17), are not uncommon and may give rise eventually to kerato-acanthomas (Fig. 18).

Possibly the most interesting tumours were found in rats treated with MC. These were the rodent type basal cell carcinomas, the tricho-epitheliomas and the sebaceous gland tumours. The division of these tumours into three types is somewhat artificial since they are all examples of basal cell tumours showing differing degrees of differentiation, the degree of differentiation being possibly dependent upon their site of origin. Nevertheless the striking differences in histological structure in well differentiated examples amply justify their individual classification.

Two basic modes of origin of these tumours were observed. In many instances the first evidence of basal cell proliferation occurred in the deeper layers of the epidermis and consisted of small downgrowths of cords and columns of basal cells associated with a mild fibroblastic reaction around their periphery (Fig. 19). These cell masses enlarged and eventually formed tumours involving the epidermis with or without surface ulceration. Another mode of development was observed in which basal cells of the pilo-sebaceous apparatus proliferated forming small groups of basal cells within the corium in intimate relationship to the remains of a hair follicle (Fig. 20). Tumours of this type frequently showed differentiation into tricho-epitheliomatous and sebaceous forms. The origin of tumours in this way from the hair follicles within the corium also explains why ulceration occurred late and why frequently there was a layer of intact epidermis and compressed fibrous tissue covering tumours of this type.

Changes in the epidermis, consisting of the development of a prickle cell layer, disorientation of cells, numerous mitotic figures and hyperchromatic nuclei were seen in many animals (Fig. 21 and 22). These changes bore a close resemblance to intra-epidermal carcinoma (Bowen's disease) of man except that there was only scanty evidence of an inflammatory infiltrate in the adjacent tissues. There is a possibility that the few examples of infiltrating squamous cell carcinoma seen in the present material were derived from these lesions, which unfortunately were unrecognisable grossly during the life of the animal, but from the appearances of the few squamous cell carcinomas observed, it appears more likely that they arose from malignant change superimposed on a pre-existing kerato-acanthoma.

It is interesting to note the differences in tumour inducing properties of the two carcinogens used in these experiments. It is clear that in this respect, with DMB the skin is less sensitive to the carcinogen than the breasts, which develop tumours one to two months before skin tumours are observed. With MC applied to the skin, breast tumours are rare ; this is in marked contrast to the results obtained when it is given orally (Huggins, Briziarelli and Sutton, 1959) ; however the skin eventually does develop tumours although some 9–10 months later than with DMB.

The reasons for the differences in tumour morphology between animals treated with MC and DMB is not known, but it may be of significance that the effect of DMB on the skin in causing epilation, with consequent greater damage to hair follicles is always greater than with MC, regardless of solvent and solution strength. This may also partly explain the differences in induction times of the tumours induced by the two carcinogens.

Whatever the reasons for these various differences the experiments described show conclusively that rats produce a wide range of skin tumours in response to carcinogenic hydrocarbons. The high incidence of basal cell carcinomas and related tumours in the animals treated with MC is of particular interest in that it offers a means for the experimental study of these common but poorly understood tumours.

### Summary

1. Experiments are described which show that rats produce a variety of skin tumours when carcinogenic hydrocarbons are applied to the skin.

2. The skin tumours fall into 2 main groups, kerato-acanthomas, and basal cell carcinomas. The latter have been subdivided for descriptive purposes into 3 sub-groups, rodent type basal cell carcinomas, tricho-epitheliomas and sebaceous gland tumours. Kerato-acanthomas were much more common in DMB-treated animals and the basal cell carcinomas and subgroups were more common in the MC-treated group.

3. The mode of development of these tumours is briefly discussed and illustrated.

My thanks are due to the Birmingham Branch of the British Empire Cancer Campaign and to the United Birmingham Hospitals Endowment Research Fund for support.

### REFERENCES

BACHMANN, W. E., COOK, J. W., DANSI, A., DEWORMS, C. G. M., HASLEWOOD, G. A. D., HEWITT, C. L. AND ROBINSON, A. M.—(1937) *Proc. roy. Soc.* B., **123**, 343.

BIELSCHOWSKY, F.—(1946) *Brit. J. exp. Path.*, **27**, 54.

GHADIALLY, F. N.—(1958) *J. Path. Bact.*, **75**, 441.—(1959) *Ibid.*, **77**, 277.—(1960) *Brit. J. Cancer*, **14**, 212.—(1961) *Cancer*, **14**, 801.

HOWELL, J. S.—(1959) *Acta. Un. int. Cancr.*, **15**, 163.

HUGGINS, C., BRIZIARELLI, G. AND SUTTON, H.—(1959) *J. exp. Med.*, **109**, 25.

LENNOX, B.—(1955) *Brit. J. Cancer*, **9**, 631.

MACCORMAC, H. AND SCARFF, R. W.—(1936) *Brit. J. Derm.*, **48**, 624.

RIGDON, R. H.—(1956) *Cancer Res.*, **16**, 804.

ZACKHEIM, H. S., SIMPSON, W. L. AND LANGS, L.—(1959) *J. invest. Derm.*, **33**, 385.